# Trend in female genital mutilation and its associated adverse birth outcomes: A 10-year retrospective birth registry study in Northern Tanzania

**DOI:** 10.1371/journal.pone.0244888

**Published:** 2021-01-06

**Authors:** Issa Rashid Suleiman, Eusebious Maro, Benjamin C. Shayo, Julius Pius Alloyce, Gileard Masenga, Michael J. Mahande, Bariki Mchome

**Affiliations:** 1 Department of Obstetrics and Gynaecology, Kilimanjaro Christian Medical University College, Moshi, Tanzania; 2 Department of Obstetrics and Gynaecology, Kilimanjaro Christian Medical Centre, Moshi, Tanzania; 3 Department of Oncology, Kilimanjaro Christian Medical Centre Moshi, Kilimanjaro, Tanzania; 4 Department of Epidemiology & Biostatistics, Institute of Public Health, Kilimanjaro Christian Medical University College, Moshi, Tanzania; University of Liverpool, UNITED KINGDOM

## Abstract

**Background:**

Approximately 200 million women and girls were reported to have undergone female genital mutilation worldwide in 2015.UNICEF’s data based on household survey estimates 15% of women from 15–49 years have undergone FGM from year 2004–2015. Despite this, reliable data on trend of prevalence of female genital mutilation and its associated birth outcomes have not been documented in Tanzania. This study aimed at determining the trends of female genital mutilation and associated maternal and neonatal adverse outcomes in northern Tanzania.

**Methods:**

A cross-sectional study was conducted using maternally-linked data from Kilimanjaro Christian Medical birth registry involving 30,286 women who gave birth to singletons from 2004–2014. The prevalence of female genital mutilation was computed as proportion of women with female genital mutilation yearly over 10 years. Odds ratios with 95% confidence intervals for adverse birth outcomes associated with female genital mutilation were estimated using multivariable logistic regression model.

**Results:**

Over the 10-year period, the prevalence of female genital mutilation averaged 15.4%. Female genital mutilation decreased from 23.6% in 2005 to 10.6% in 2014. Female genital mutilation was associated with increased odds for caesarean section (aOR1.26; 95% CI: 1.18–1.34), post-partum haemorrhage (aOR 1.31; 95% CI: 1.10–1.57) and long hospital stay (aOR 1.21; 95% CI: 1.14–1.29). Female genital mutilation also increased women’s likelihood of delivering an infant with low Apgar score at 5^th^ minute (aOR 1.60; 95% CI: 1.37–1.89).FGM type III and IV had increased odds of caesarean section, episiotomy and prolonged duration of hospital stay as compared to FGM type I and II, although the association was statistically insignificant.

**Conclusion:**

Female genital mutilation prevalence has declined over the study period. Our study has demonstrated that postpartum haemorrhage, delivery by caesarean section, long maternal hospital stays and low APGAR score are associated with FGM. Initiatives to mitigate FGM practice should be strengthened further to reduce/eliminate this practice. Moreover, surgical interventions to improve severe form FGM are welcomed to improve the aforementioned aspects of obstetric outcome in this locality.

## Introduction

Female genital mutilation (FGM) constitutes a global health concern being practiced in more than 30 countries in Africa, Asia and the Middle East [1]. Despite report of at least 200 million FGM cases in 30 countries (from the year 1985–2016) the’ exact magnitude of FGM remains unclear [1].

Globally, the prevalence of FGM has been reported to decline over the past three decades, except in the sub Saharan Africa. Among the 30 countries which reported to practice FGM, the highest prevalence was observed in Somalia (98%), Guinea (97%), Djibouti (93%), Yemen (21%), Kenya (19) and Tanzania (15%) [1]. Despite this, concern prevail regarding accuracy of the reported magnitude of due to underreporting given its illegality [2].

FGM has been associated with obstetrics complications, but there is limited evidence and controversies surrounding it [3]. For instance, a small scale prospective cohort study done in a predominantly Muslim community in Ethiopia demonstrated that women with FGM had higher odds of emergency caesarean section and postpartum haemorrhage [4]. Contrary to that, a small scale case control retrospective study done in London showed no significant difference in blood loss post-delivery nor in the rate of emergency caesarean delivery [5]. These could be attributed to the difference in the quality of obstetric care with intrapartum defibulation variation in practise.

A multi-centre study on neonatal complications among women with FGM revealed no significant differences in proportion of infants with low Apgar score between mothers with FGM compared to their counterparts [6]. Contrary to this, an observational study in Burkina Faso foetus delivered with Apgar scores of 9/10 were lower among women with FGM compared to their counterparts (17% vs. 51%), respectively [7]. These controversies call for further studies to have clear evidence. Data on the burden of FGM and its associated adverse birth outcomes in low and middle income countries are sparse [6].

According to Tanzanian Special Provision Act, a 1998 amendment to the penal code specifically prohibits Female Genital Mutilation/Cutting (FGM/C). Despite this, FGM is still prevalent in many areas in Tanzania [8]. Previous authors have showed that, approximately 7.9 million women and girls have undergone FGM in Tanzania for the past 2 decades [9]. The prevalence of FGM among women of reproductive age (15–49) in Northern regions is still unacceptably high i.e. Manyara (58%), Arusha (41%) and Kilimanjaro (10%) [10].

In an analytical study done by Abdulcadir et al., they reviewed available literature about the clinical care of women with FGM in identifying the existing evidence based and research gaps. They showed that the impact of healthcare quality and use on obstetric outcomes such as rates of caesarean delivery, postpartum hemorrhage, extended hospital stay, and neonatal outcomes is unknown [11].

The current study took advantage of existing large scale database to explore the trend of FGM and associated adverse birth outcome among women with singleton delivery in Northern Tanzania. Previous studies in Tanzania used small scale, and mainly focused on attitude and social correlation of the FGM [12], while some explored on the impact of long campaigns which were conducted to eradicate or reduce FGM practice [13]. Thus, little has been documented on the effect of FGM on birth outcomes in Tanzania. This study aimed to determine the trend in female genital mutilation and associated maternal and neonatal adverse outcomes in northern Tanzania.

## Material and methods

### Study design and setting

This was a cross-sectional study which was designed to use maternally-linked data from Kilimanjaro Christian Medical birth registry for women who delivered singletons from 2004–2014 at Kilimanjaro Christian Medical Centre (KCMC). KCMC is a referral and teaching hospital with 1200 inpatients in 630 official beds located in Northern zone of Tanzania, Kilimanjaro region. It serves 4 regions of the Northern Zone-Tanzania including Kilimanjaro, Arusha, Tanga and Manyara. The population of Kilimanjaro region is estimated to be 1,640,087 and at least 6,804,733 in all the four regions [14]. The KCMC medical birth registry has been operating since 2000 with 70,000 deliveries recorded since its inception to December 2019. Maternity care cost at KCMC is provided in subsidised cost. Majority of client are not covered by health insurance pays privately or through a social welfare scheme. In this locality there is no specialised antenatal care for women with FGM.

### Study population and sampling procedure

**Fig 1:**
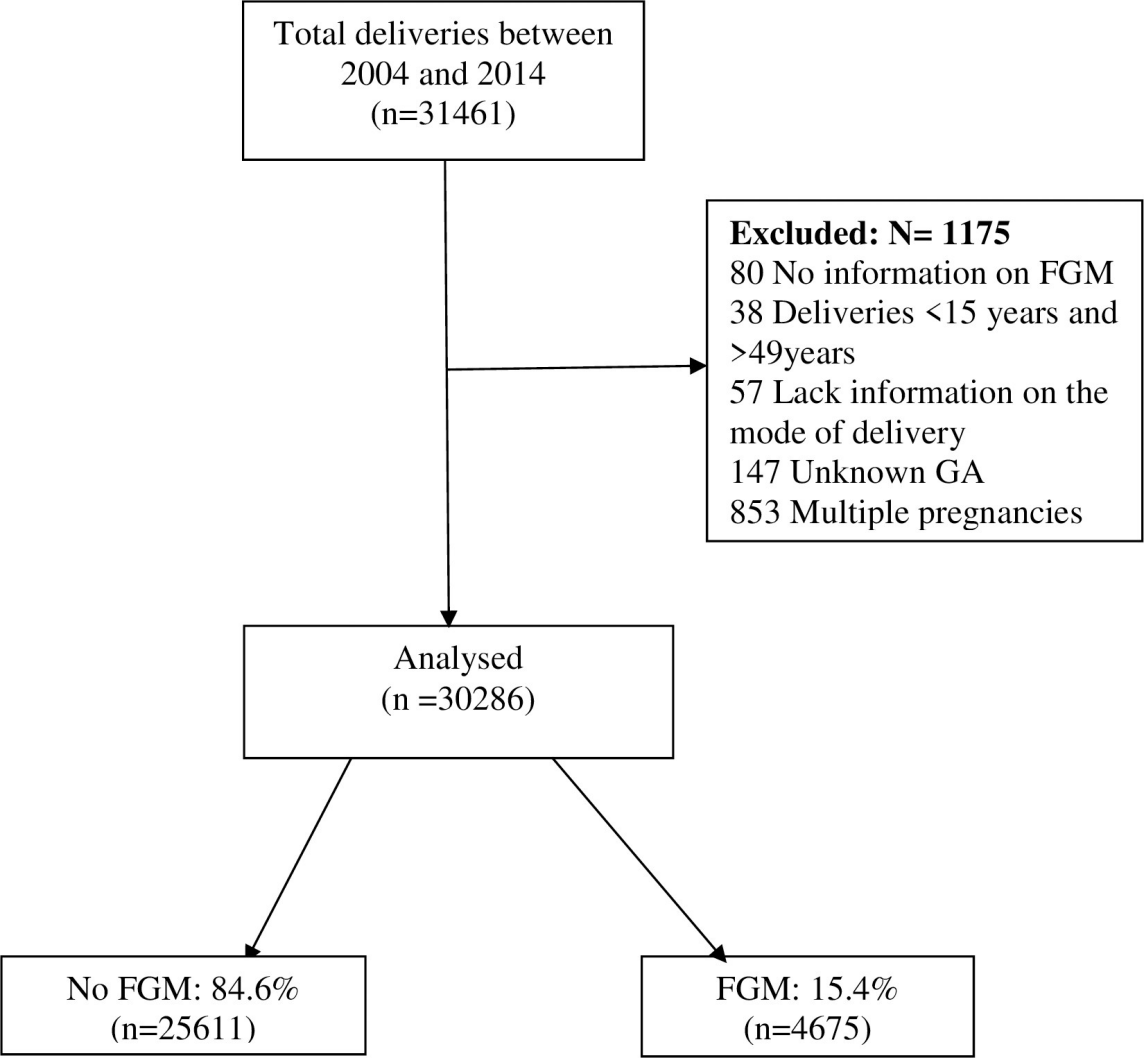
Enrolment of participants.

This study enrolled all women who delivered at KCMC hospital between 2004 and 2014. Inclusion criteria included singleton women aged 15–49 years. We excluded multiple gestations, missing records on FGM status, missing records on gestation age at delivery and those with missing records on the mode of delivery. The final sample size was 30,286 women “Fig 1”.

### Data collection method and tools

Data extraction sheet was used to obtain information on socio-demographic characteristics, obstetric history, FGM status and immediate maternal and foetal complications from the medical birth registry database. The status on FGM was recorded from direct observation by the attending midwife and documented in the partograph during the routine examination in labour and delivery. Trained midwife nurses conduct interviews on a daily basis using a standardized questionnaire of the hospital. Mothers who were admitted were also asked to provide their antenatal cards from which relevant information was abstracted and then all this information is entered in the KCMC medical birth registry database.

### Study variables and definition

The primary outcome of interest included caesarean delivery, episiotomy, postpartum haemorrhage (PPH) and prolonged duration of hospital stay. In our study, the latter was defined as staying in hospital for more than 24hours after vaginal delivery and/or for more than 72hours after caesarean delivery. And PPH was defined as bleeding of approximately >500mls after vaginal delivery or/and >1000mls after caesarean section delivery. Other outcomes included, low APGAR score which was defined as a score of less than 7 in the fifth minute of a new born. And early neonatal death which was defined as death of a new born baby, within 24 hours of life.

FGM was the main exposure variable in our study which is defined as partial or total removal of external genitalia or other injury to the female genital organs for non-medical reasons. Only women with documented FGM on the birth registry database were selected. According to WHO, FGM is classified in type I, II, III and IV. Type I consists of the removal of the prepuce, with or without the excision of the clitoris Type II consists of the removal of the clitoris with partial or total excision of the labia minora. Type III involves the removal of a part or the whole external genitalia, making a suture of the vaginal canal (also called ‘‘infibulation”). Type IV is unclassified and includes all the procedures modifying normal external genitalia anatomy (e.g., drilling, piercing, cutting, clitoris cauterization, vaginal orifice abrasion) [15].

Other covariates include socio-demographic characteristics such as maternal age, level of education, marital status, occupation, residence, tribe, residency, religion, gestation age at delivery, and parity. Other exposures included complications during pregnancy such as antepartum haemorrhage (APH), which is bleeding between 28 weeks of gestation to delivery. Preeclampsia, gestational diabetes mellitus (GDM), Diabetes type II in pregnancy, anaemia and body mass index (BMI). BMI was categorised into 4; underweight, normal weight, overweight and obese.

### Statistical analysis

Data was abstracted from Microsoft access and then sorting, cleaning and checking for consistency and duplicates followed by data analysis using STATA (Version 13.0). Descriptive statistics were summarized using frequency and proportions for categorical variables while mean and standard deviation (SD) were used for numerical. Trend in prevalence of FGM was computed as a proportion of female who underwent FGM practice during the study period. Chi-square test was used to determine the association between FGM and a set of medical conditions in a bivariate analysis. Both crude and adjusted odds ratios and 95% confidence interval for adverse birth outcomes associated with FGM with were estimated using multivariable logistic regression models. A p-value of <0.05 was considered statistically significant.

### Ethical considerations

Ethical approval no. 2323 was obtained from the Kilimanjaro Christian Medical University College Research Ethics Committee prior to starting data collection. Permission was sought from the department of Obstetrics and Gynaecology at KCMC Hospital. No informed consent was sought from clients as their consent to store their records in the birth registry allows the analysis and reporting of their anonymous records for research purposes. Access to this information was made available only to the researcher, the assistant and supervisors.

## Results

### Enrolment of participants

A total of 31,461 women delivered at KCMC between 2004 and 2014. Among them, 1,175 women did not meet the eligibility criteria and were excluded from the study remaining 30,286 women whose records were analysed “Fig 1”.

### Socio-demographic and obstetric characteristics of women delivered

The overall mean age of the participants was 27.7± 6.2 years with those reporting FGM being slightly older as compared with their counterparts 29.4±6.7 vs. 27.4 ±6.0 years, respectively. Majority of the participants were married, had primary education, self-employed and were primigravida (Table 1).

**Table 1:** Socio-demographic and obstetric characteristics (N = 30286).

Characteristics	No FGM (n = 25611)	FGM (n = 4675)	p-value
**Age in years**			<0.0001
<20	2299(9.0)	364(7.8)	
20–24	6756(26.6)	913(19.5)	
25–29	7635(29.8)	1063(22.7)	
30–34	5417(21.2)	1154(24.7)	
≥ 35	3504(13.7)	1181(25.3)	
[Mean, SD]	[27.4,6.0]	[29.4, 6.7]	
**Marital status**			<0.0001
Married	21775(85.0)	4290(91.8)	
Unmarried	3836(15.0)	385(8.2)	
**Education**			<0.0001
None	307(1.2)	295(6.3)	
Primary (std 1–7)	12846(50.2)	3402(72.8)	
Secondary(form1-4)	3956(15.4)	326(7.0)	
Higher education	8502(33.2)	652(13.9)	
**Occupation**			<0.0001
Housewife	4487(17.5)	986(21.1)	
Self-employed	14633(57.1)	3274(70.0)	
Employed	5602(21.9)	378(8.1)	
Students	889(3.5)	37(0.8)	
**Tribe**			<0.0001
Chagga	14410(56.3)	1324(28.3)	
Pare	2810(11.0)	1137(24.3)	
Maasai	208(0.8)	352(7.5)	
Others	8183(32.0)	1862(39.8)	
**Residency**			<0.0001
Rural	11859(46.3)	2942(62.9)	
Urban	13752(53.7)	1733(37.1)	
**Religion**			<0.0001
Christians	2067680.7)	3039(65.0)	
Muslims	4484318.9)	1616(34.6)	
Others	92(0.4)	20(0.4)	
**Referral status**			<0.0001
Self-referred	19346(75.5)	2829(60.5)	
Referred	6265(24.5)	1846(39.5)	
** Gestation age**			<0.0001
Preterm	6209(24.2)	1463(31.3)	
Term	19402(75.8)	3212(68.7)	
** Parity**			<0.0001
Primipara	11398(44.5)	1359(29.1)	
Multipara	6659(26.0)	1029(22.0)	
Grandmultipara	6655(26.0)	2177(46.6)	
Unknown	899(3.5)	110(2.4)	
** Mode of delivery**			
Vaginal	16607(64.9)	2782(59.4)	<0.0001
Caesareans section	8766(34.2)	1854(39.6)	
Vaccum	230(0.9)	47(1.0)	
Birth weight			<0.0001
<2.5kg	2514(9.8)	602(12.9)	
2.5–3.9kg	21961(85.7)	3892(83.3)	
>4kg	1136(4.4)	181(3.9)	
[Mean; SD]	[3.1; 0.5]	[3.0; 0.6]	
** Stillbirth**			
No	24886(97.2)	4440(95.0)	<0.0001
Yes	725(2.8)	235(5.0)	

### Medical conditions during pregnancy

More than half (56.7%) of the participants had normal BMI and 10.4% were obese. There was significant difference between women with and without FGM in respect to BMI, APH and anaemia (Table 2).

**Table 2:** Medical conditions during pregnancy (N = 30286).

Variables	No FGM (n = 25611) n(%)	FGM (n = 4675) n(%)	χ^2^-P-value
**BMI group**			0.040
<18.5	549(2.1)	86(1.8)	
18.5–24.9	14475(56.5)	2693(57.6)	
25.0–29.9	7879(30.8)	1458(31.2)	
≥ 30	2708(10.6)	438(9.4)	
**DM in pregnancy**			0.392
No	25538(99.7)	4665(99.8)	
Yes	73(0.3)	10(0.2)	
**Preeclampsia**			0.315
No	24564(95.9)	4469(95.6)	
Yes	1047(4.1)	206(4.4)	
**APH**			0.001
No	25251(98.6)	4578(97.9)	
Yes	360(1.4)	97(2.1)	
**Anaemia**			0.009
No	25157(98.2)	4617(98.8)	
Yes	454(1.8)	58(1.2)	

### Prevalence and types of female genital mutilation

**Fig 2:**
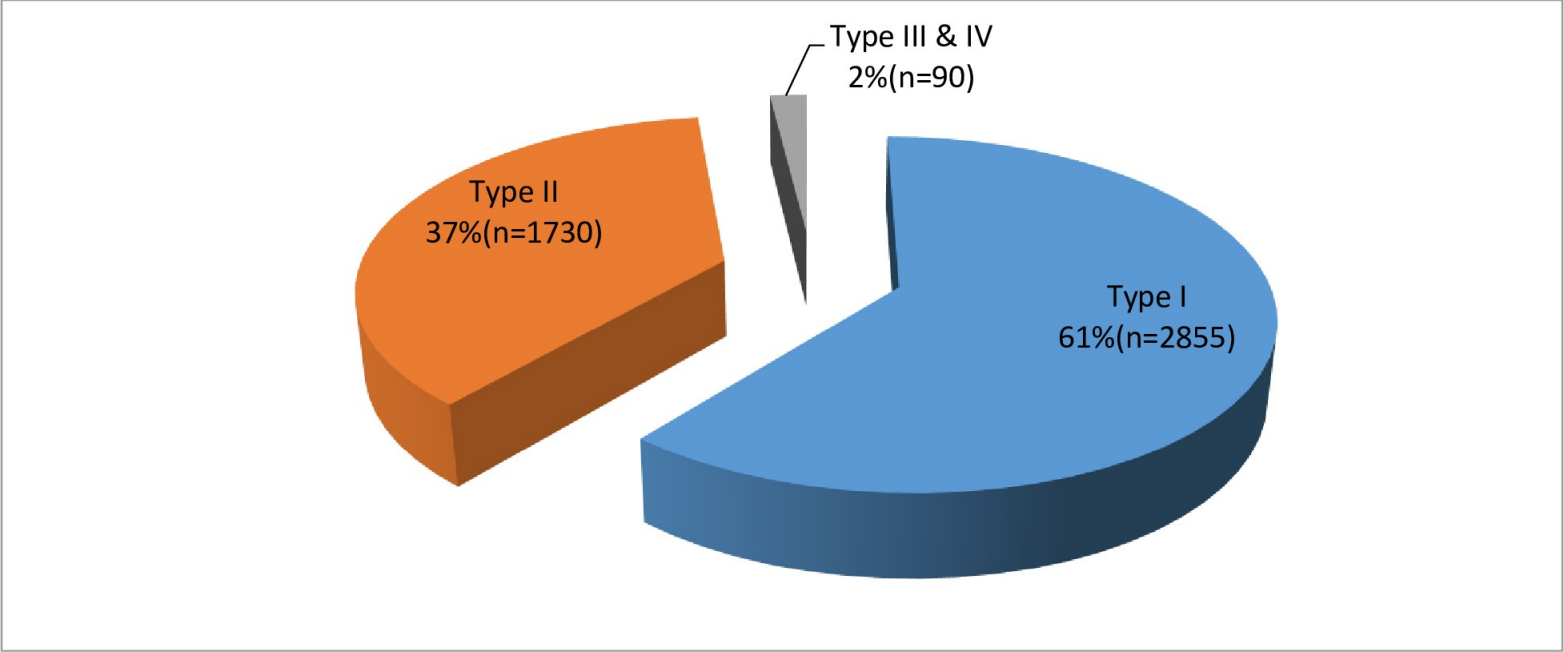
Proportion of female genital mutilation by type.

The general prevalence of FGM was 15.4%. Of 4675 women who reported to have undergone FGM, nearly two-third (61%) had female genital mutilation type I, followed by type II (37%). The type III and IV were infrequently practiced (2%) **“**Fig 2”.

### Trend in female genital mutilation

**Fig 3:**
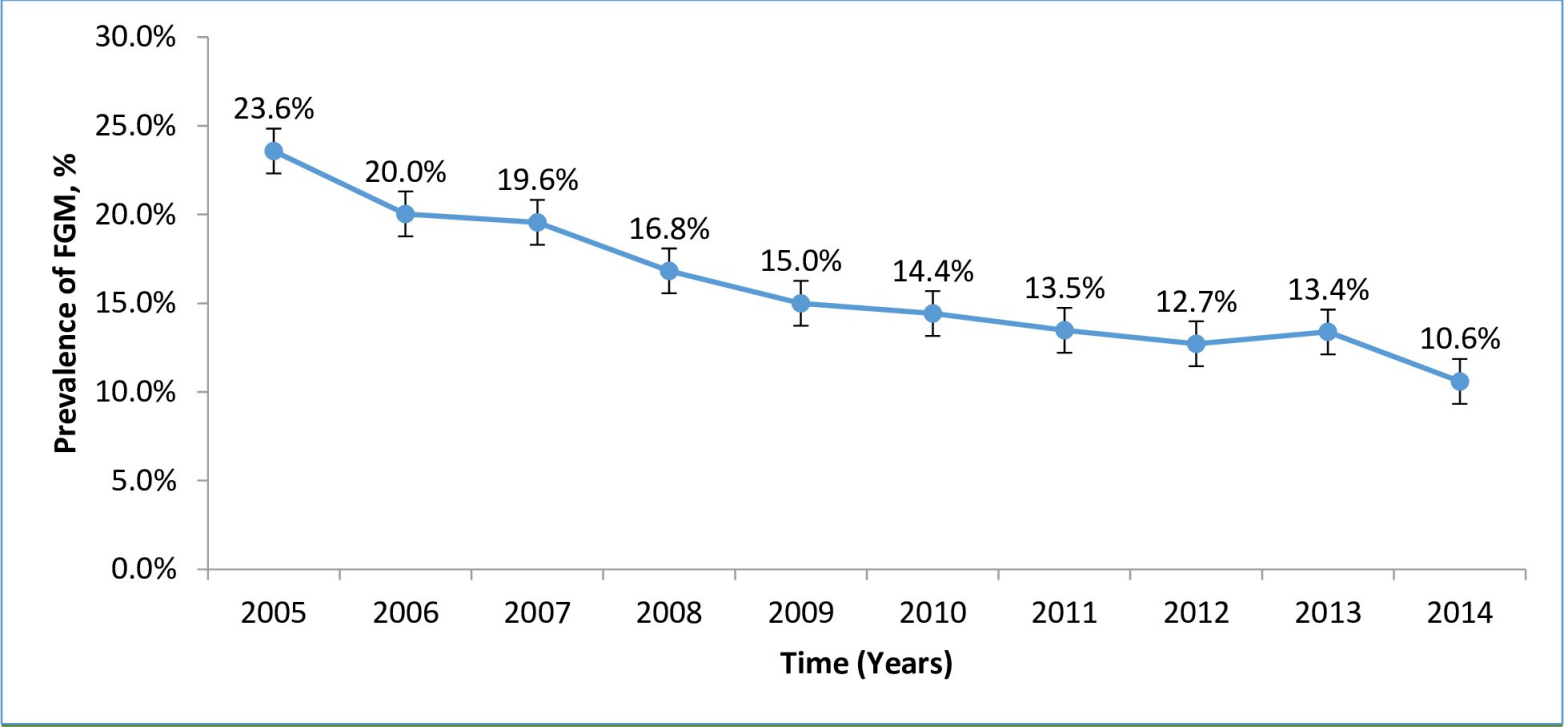
Trend in prevalence of female genital mutilation.

The trend in prevalence of FGM is shown in “Fig 3”. The proportion of women who underwent FGM declined from 23.6% in 2005 to 10.6% in 2014. This decline was statistically significant with an average decrease of 1.3% per year (χ^2^ = 293.48, p<0.0001) with no overlapping of confidence interval between 2004 and 2014.

### Adverse maternal outcomes associated with female genital mutilation

The result from a bivariable logistic regression analysis showed that that women who underwent FGM had significantly increased odds of having caesarean delivery [OR 1.26 (95% CI: 1.18–1.34)], PPH [OR 1.31(95% CI: 1.10–1.57)], and long hospital stay [OR 1.21(95% CI: 1.14–1.29)]. Women who had FGM type III and IV had the most risk for adverse maternal outcomes compared with their counterparts who had type I & II. However, this association was not statistically significant (Table 3).

**Table 3:** Maternal adverse outcomes associated with FGM (N = 30286).

Variables	Complications			
	Caesarean Delivery	Episiotomy	PPH	Long hospital stay
**Bivariable (cOR)**				
FGM	1.26(1.18–1.34)	1.08(0.92–1.27)	1.31(1.10–1.57)	1.21(1.14–1.29)
**FGM type**				
Type I	1.24(1.15–1.34)	1.36(1.14–1.63)	1.23(0.98–1.52)	1.13(1.04–1.22)
Type II	1.20(1.09–1.33)	0.68(0.51–0.92)	1.35(1.04–1.76)	1.30(1.17–1.43)
Type III&IV	1.62(1.07–2.46)	0.57(0.14–2.30)	1.69(0.62–4.62)	1.28(0.84–1.95)
**Multivariable (aOR)**				
FGM	1.20(1.13–1.28)	1.30(1.11–1.53)	1.11(0.93–1.33)	1.20(1.13–1.28)
**FGM type**				
Type I	1.16(1.08–1.26)	1.76(1.46–2.11)	1.02(0.81–1.27)	1.12(1.03–1.21)
Type II	1.19(1.08–1.32)	0.72(0.53–0.97)	1.22(0.93–1.60)	1.28(1.16–1.42)
Type III&IV	1.50(1.00–2.27)	0.73(0.17–3.09)	1.41(0.53–3.71)	1.25(0.83–1.90)

cOR- Crude Odds Ratio; aOR- Adjusted Odds Ratio (maternal age, gestational age, parity, BMI, APH, Anaemia).

In a multivariable logistic regression analysis, FGM remained an important predictor for caesarean delivery [aOR (1.20 (95% CI: 1.13–1.28)], episiotomy [aOR (1.30 (95% CI: 1.11–1.53)], and long hospital stay [aOR (1.20 (95% CI: 1.13–1.28)], after adjusting for maternal age, gestational age, parity, APH, anaemia and BMI. In addition, women with FGM had 1.11 higher risk of PPH during delivery, although this was not statistically significant [aOR 1.11(95% CI: 0.93–1.33)]. Like in, FGM types (III &IV) showed risk of these adverse maternal outcomes but there was no statistical evidence (Table 3).

### Adverse neonatal outcomes associated with female genital mutilation

The result from the bivariate logistic regression analysis revealed that, women who underwent FGM had an increased odd of delivering an infant with low Apgar score at 5^th^ minute [cOR (1.60 (95%CI: 1.37–1.89)]. The FGM also increased women’s likelihood of losing their babies during perinatal period [cOR (95%CI): 1.12(0.69–1.81)].

Furthermore, when analysed by FGM type, in a crude analysis, women who had types III and IV had more than 3-folds higher odds of delivering an infant with low Apgar score [cOR 3.36 (95% CI: 1.62–6.98)]. Similarly, these types were associated with higher odds of early neonatal death, although this association was not statistically significant [cOR (2.89 (95% CI: 0.40–20.91)]. FGM type I and II were also significantly associated with low Apgar score [cOR (1.30 (95%CI: 1.05–1.60) and [cOR (1.81(95%CI: 1.44–2.28)] respectively. The association with early neonatal death did not reach statistical significance.

In the multivariable logistic regression model, FGM remained an important predictor for low Apgar score [aOR 1.32 (95% CI: 1.11–1.56)]. Furthermore, FGM type II and the combined types III and IV were significantly associated with an increased odds of low Apgar score [aOR 1.47 (95% CI: 1.16–1.88] and [aOR (3.22 (95% CI: 1.42–7.30)] respectively. The individual type of FGM was not significantly associated with neonatal death. However, the combined type III and VI had more than 2-fold increased odds of having of neonatal death [aOR (2.30 (95% CI: 0.31–17.30)], although, this was not statistically significant (Table 4).

**Table 4:** Neonatal adverse outcomes associated with FGM (N = 30286).

	Complications	
Variables	Low APGAR score	Early neonatal death
**Bivariable(cOR)**		
FGM	1.60 (1.37–1.89)	1.12(0.69–1.81)
**FGM type**		
Type I	1.30 (1.05–1.60)	0.79(0.40–1.57)
Type II	1.81 (1.44–2.28)	1.53(0.80–2.93)
Type III& IV	3.36(1.62–6.98)	2.89(0.40–20.91)
**Multivariable(aOR)**		
FGM	1.32(1.11–1.56)	0.94(0.57–1.55)
**FGM type**		
Type I	1.10(0.88–1.36)	1.09(0.97–1.23)
Type II	1.47(1.16–1.88)	1.24(0.64–2.41)
Type III& IV	3.22(1.42–7.30)	2.30(0.31–17.30)

cOR- Crude Odds Ratio; aOR- Adjusted Odds Ratio (adjusted for maternal age, gestational age at delivery, parity, APH, Preeclampsia, GDM, Diabetes type II in pregnancy, Anaemia and birth weight).

## Discussion

The results from this study showed a decline in trend of FGM over the past 10 years from 23.6% in 2005 to 11.6% in 2014; an average decrease of 1.3% per year. FGM was associated with an increased use of episiotomy, delivery by caesarean section, prolonged hospital stay, and low Apgar score.

The observed decline in the trend of FGM prevalence in our study is similar to the previously reported in Egypt of 67.6% in 2005 to 57.8% in 2014 [16], Kenya of 32.2% in 2003 to 21% in 2014 [17], and in Ghana from 35.2% in 1996 to 21.1% in 2003 [18]. But it is different with the trend that was observed among Australian women which indicated fluctuating over the 6 years period [19]. The observed difference might be because, FGM in Australia and its diaspora is observed mainly among migrants, therefore FGM prevalence depends on the number of migrants that entered those nations over time. The progressive decline in prevalence of FGM in our study population might be due to the continuous health risk campaigns about the health consequences of FGM for the girls and the women, recruitment of change agents from within the communities and the enforcement of legal mechanisms by the government of Tanzania and other health stakeholders [13].

Our study found that FGM was significantly associated with immediate maternal adverse outcomes. Women who were exposed to FGM had a 1.26 higher risk of delivery by caesarean section than the non-exposed group, although, none of the caesarean sections had FGM sorely as an indication for the operation. This finding is consistent previous studies in Ethiopia [4] and Australia [19]. But, it is inconsistent with the study that was done in the united Kingdom (London) which showed no difference in the caesarean section rate among women with and without FGM [5]. This difference might be due to the differences in the sample sizes and the standard of care. Compared to our study’s sample size of 30,286 women, 242 participants in the London study might have been too little to detect the statistical significance. During the antenatal period, all women with FGM are reviewed in a dedicated perineal clinic. A consultant urogynaecologist and/or a specialist perineal midwife review these patients, and an individualized plan of care is developed [5]. This level of care is not available in our setup.

In the present study, episiotomy use was higher in women who had FGM compared to those without FGM. There were 1.30 higher odds of having an episiotomy in women with FGM as compared with their no-FGM counterparts. This finding is similar to that reported in the multi-centre study among 6 African countries (Burkina Faso, Ghana, Kenya, Nigeria, Senegal and Sudan) [6] and to a study done in London [5]. Our result is different from a study done in Australia which showed no increase in episiotomy use to women with FGM [19]. The significant increase in use of episiotomies in women with FGM was expected, because FGM is associated with increased inelastic scar tissue around the introitus, which may restrict stretching of the perineum and delay the second stage of delivery [3]. Episiotomies incise scar tissue, with a resultant increase in soft tissue space, thereby expediting delivery.

Women with genital mutilation were found to have 1.20 higher odds of staying in the hospital longer after delivery compared with women without mutilation. Similar find was observed in a multi-centre study done in 6 African countries [6]. This may be because women with FGM are more likely to experience complications that warrant longer hospital stay for care.

In this study, PPH was found to have a positive association with FGM, though it was not statistically significant. But this might be of clinical importance. In our study, women with FGM had 11% higher odds of developing PPH compared to women without PPH. Similar find was reported in Ethiopia [4]. This observation might be due to the long-term complications of FGM where during healing, there might be formation of scars on the perineal and vaginal tissues. This scar tissue is less elastic and not as strong as healthy tissues. Therefore, the presence of this scarring tissue attributes to the increased bleeding from high rate of episiotomy.

FGM was found to have a significant positive association with low Apgar score. Women with FGM have 32% higher odds of delivering a baby with low Apgar score compared to those women without FGM. Our finding is in contrast with that from the multi-centre study done in 6 African countries [6] and that which was done in Djibouti [20]. This difference in results might be due to difference in study design. Our study was a retrospective cross-sectional study and therefore, we were not able to account for all the confounders for low Apgar score and this may over inflate the association observed while these other two studies were prospective cross-sectional and observational case-control studies respectively.

### Strength and limitations

To our understanding, this is a large scale study with the ability to capture linked maternal and fetal data among women with FGM to be conducted in East and Central Africa. Potential drawbacks inherent in retrospective investigations have similarly been encountered in the present study, including missing information like number of maternal deaths, time of death and causes of death, lack of detailed information on intrapartum care including indications for c/section and other important variables that might have some effect on the outcomes. Moreover, the types specific classification of FGM relies on rather subjective assessment that may have potentially misclassify cases between different types. Additionally, since this practise is accustomed to few ethnic groups whose distribution is quite patchy, our findings may not be generalizable. Furthermore, since this was a referral hospital-based study, our results cannot be generalized to the whole Tanzania population. We therefore recommend future multi-centre prospective studies to be done as they are more representative.

### Conclusion

This study shows that the trend in the prevalence of FGM in northern Tanzania is decreasing overtime. It also revealed FGM increases women’s likelihood to have adverse maternal (caesarean delivery, episiotomy and long hospital stay), and neonatal (low APGAR score in the fifth minute and early neonatal death) outcomes compared to women without FGM.

Our findings could be used to put more emphasize and create more awareness to the communities on the harmful effects of FGM that might lead to more decrease of this practise and/or elimination.
